# “It's a reality brought to us by the patients”: a qualitative study on general practitioners’ views on and experience with complementary medicine in Switzerland

**DOI:** 10.1186/s12875-025-02999-4

**Published:** 2025-10-17

**Authors:** Julie Dubois, Pierre-Yves Rodondi, Christina Akre

**Affiliations:** 1 Institute of Family Medicine, Faculty of Science and Medicine, University of Fribourg, Fribourg, Switzerland; 2 Faculty of Biology and Medicine, University of Lausanne, Lausanne, Switzerland; 3 Department of Epidemiology and Health Systems, Center for Primary Care and Public Health (Unisanté), University of Lausanne, Lausanne, Switzerland

**Keywords:** Primary care, Complementary medicine, General practice, Qualitative research

## Abstract

**Background:**

A significant proportion of general practitioners (GP) practice, recommend or prescribe complementary medicine (CM) to their patients. However, so far, little is known about their prescribing and referral habits, as well as their needs and expectations regarding CM in everyday practice. This study aimed at exploring how CM is viewed and experienced by GPs in clinical practice in Switzerland.

**Methods:**

This descriptive qualitative study was conducted among GPs with no additional training in CM and working in private practices. Data were collected through semi-structured interviews based on an interview guide reflecting the study objectives. The verbatim transcripts of the interviews were coded using a thematic analysis approach.

**Results:**

The analysis generated four main themes: perceptions of CM; recommending CM; discussing CM during the consultation; and needs for the future. Overall, most participants had a positive attitude towards CM, coupled with cautiousness and skepticism when faced with the diversity of therapies. Both perceptions of and recommendations for CM were largely influenced by GPs’ personal knowledge, experiences and beliefs. Knowledge about CM, belief in efficacy, availability of scientific evidence, and trust in CM therapists were all facilitators to CM recommendation. Lack of knowledge about CM, lack of insurance coverage, and lack of network were barriers to CM recommendation. During consultations, the subject was more often tackled by patients. However, GPs generally encouraged their patients in their care pathway if they felt that their patients’ CM use would not harm them. Needs for the future revolved around training and information sources. GPs advocated introductory courses about CM, in post-graduate or continuous education. In addition, GPs underlined the need for a tool that would centralize information on CM and be easily accessible.

**Conclusion:**

GPs in our study tend to have a pragmatic approach to CM where not only scientific evidence is considered, but also patient’s wishes and GPs’ own experience and beliefs. Above all, GPs are concerned that patients should be safe and well cared for. Future initiatives to further integrate CM into the healthcare system should thus focus on providing courses and informational tools to GPs to allow them to better support and advise their patients.

**Supplementary Information:**

The online version contains supplementary material available at 10.1186/s12875-025-02999-4.

## Background

Complementary medicine (CM) is defined by the World Health Organization as “a broad set of health care practices that are not part of that country’s own tradition or conventional medicine and are not fully integrated into the dominant health-care system” [1]. Multiple studies have highlighted the widespread use of CM by the population in western countries in the last decade [2]. Most CM users use it alongside conventional medicine [3, 4] and call for a better integration of CM within conventional care [5, 6].

As first-line providers of care and gatekeepers for access to a variety of both medical and nonmedical services, GPs are well-positioned to play a role in the integration of CM within the conventional healthcare system [7, 8]. In the US, CM is recommended or prescribed in 4.9% of GPs consultations [9]. Furthermore, recent surveys have shown high proportions of GPs practicing, recommending or prescribing CM, ranging from 63 to 88% [10–12].

Several recent studies have investigated the interest of GPs for CM and their use thereof in their everyday medical practice [11, 13–20]. This interest could be partly due to the fact that many recommendations for CM concern pathologies that are common in primary care (low back pain, fatigue, etc.) [3, 11, 17]. Commonly cited reasons for practicing or recommending CM are belief in efficacy, adopting a more holistic approach, lack of response to conventional treatments, and consideration of patient wishes [17, 21, 22]. Nonetheless, existing studies point to numerous barriers to the integration of CM in primary care, including lack of network and knowledge of the scope of practice of CM therapists, lack of scientific evidence and issues around (non-)reimbursement of care [11, 17, 18, 22].

Switzerland constitutes an interesting setting to investigate how CM is integrated into GPs practice. Indeed, in Switzerland, physicians can obtain postgraduate certificates in four CM methods (homeopathy, herbal medicine, acupuncture, anthroposophic medicine). In addition, in 2017, CM was introduced in the learning objectives for medical students (Principal Relevant Objectives and Framework for Integrative Learning and Education in Switzerland – PROFILES) and courses on CM have been mandatory in medical schools since then [23]. Non-physician CM therapists are not formally regulated by law in the country, but federally recognized diplomas in a variety of CM techniques and approaches have been in place since 2015. However, these diplomas are not yet mandatory to be able to practice CM. Regarding reimbursement, CM treatments delivered by physicians holding a postgraduate certificate in CM are covered by the Swiss mandatory health insurance. Some herbal medicine treatments are also covered when prescribed by any physician. Other CM treatments, or treatments performed by non-physician therapists, are covered by supplemental insurances. Finally, the most recent survey on CM use in Switzerland showed that 29% of the population aged 15 or more had used CM at least once in the past 12 months, with 75% of CM users also consulting with a GP over the same period [24].

Despite a high percentage of concomitant use of CM and general medicine, so far, little is known about GP’s prescribing and referral habits, as well as needs and expectations regarding CM in everyday practice. This study aimed at exploring how CM is viewed and experienced by GPs in clinical practice in Switzerland.

## Methods

### Study design

This descriptive qualitative study [25] was conducted among GPs by means of semi-structured interviews. A qualitative approach was chosen because it allows to explore experiences and obtain in-depth information from the study participants.

### Study population

The study population consisted of GPs working in private practices in three French-speaking cantons in Switzerland. To be included, GPs had to meet the following inclusion criteria: 1) hold a specialist title in general internal medicine; 2) to currently be working in a private practice in the cantons (states) of Vaud, Fribourg or Neuchâtel; 3) be willing to participate in a single semi-structured interview conducted in French. GPs holding a postgraduate certificate of additional training in CM delivered by the Swiss Institute for medical postgraduate and continuing education were excluded from the study. It is estimated that 8% of Swiss GPs hold such certifications [26]. This decision was made since these GPs may hold more positive views on CM than GPs without such training, thus potentially biasing our sample.

### Data collection

An interview guide was elaborated based on the study objectives, on discussions with colleagues working as GPs and on a review of qualitative research conducted on the subject [11, 13–20]. It was pilot tested with a GP to ensure its comprehensibility and coherence (See Additional file 1).

Recruitment was conducted using convenience, purposive and snowball sampling techniques. Recruitment was guided by a maximum variation sampling strategy to ensure representativeness of GPs. To do so, a sampling grid with predefined socio-demographic criteria was established, including age, gender, and canton of practice. Attention was paid during recruitment to achieve balance in GP distribution within each of the sampling grid criteria. Recruitment was made through our own network of teaching GPs. The authors directly contacted 50 GPs and also asked their colleagues at the Institute of family medicine to disseminate the invitation to participate in their own network. All GPs were invited to participate in the research project by e-mail with an information letter explaining the purpose and procedures of the study attached. When interested they responded to the email and were then contacted, either by phone or e-mail, to set up a date and location for the interview. Participants were also asked to inform other potentially interested colleagues about the study. Before the interviews, the authors checked that none of the participants held a postgraduate certificate of additional training in CM. Interviews took place between September 2023 and June 2024, and were conducted by JD (first author, anthropologist, senior qualitative researcher in complementary medicine and primary care). Before the interviews, JD recalled the study objectives and information contained in the information letter, notably regarding data confidentiality. All participants gave their informed oral consent to participate before the beginning of the interviews. All interviews were audio recorded and then transcribed verbatim.

### Data analysis

A thematic analysis was performed with the assistance of the qualitative data analysis software MaxQDA (v.2022.8), following the six phase process described by Braun et al. [27]. As a first step, JD familiarized herself with the data by reading all the transcripts. Second, JD and CA (last author, anthropologist, senior qualitative researcher in public health) separately coded the same transcript and compared their coding to establish a first set of codes (codebook) and to discuss and reach an agreement over the coding process. Then, the remaining transcripts were coded by JD based on the initial codebook. The latter was adapted iteratively during the analysis when new codes were created. To ensure consistency in the coding process, JD wrote memos to describe each code and its meaning. Third, all the codes were reviewed, similar ones were merged and then sorted into larger subthemes and themes. To fit our descriptive orientation, we chose a semantic approach to generate out themes, meaning that they aim to reflect the explicit meaning of the data, contrary to a more interpretive approach [27]. Fourth, all themes and subthemes were reviewed to insure they were coherent both individually and in relation to the whole data set. Fifth, themes and subthemes were further analyzed to identify the relations between different themes within each interview and across the interviews. Several meetings were held with CA and PYR (second author, primary care physician and professor in primary care) during the whole process to discuss the analysis, confirming or challenging it, in order to reach an agreement over the interpretation of the data.

The data presented in this manuscript are based on participants’ quotations. JD translated them from French to English. An additional reading was carried out by CA, a native English speaker, to make sure the idiomatic meaning of the statements was preserved. We used the Consolidated criteria for Reporting Qualitative research (COREQ) checklist to report our study [28].

## Results

A total of 14 interviews (7 with female GPs) were conducted. They took place at the GPs practice or via videoconference, depending on participants’ preferences, and lasted between 31 and 65 min (mean: 49 min). The mean age of the participants was 47.5 (Range: 38–60). Seven GPs practiced in the canton of Vaud, four in the canton of Fribourg and three in the canton of Neuchâtel. The analysis of the interviews generated four main themes: perceptions of CM; recommending CM; discussing CM during the consultation; and needs for the future.

### Perceptions of CM

#### What is CM?

Participants underlined that the term CM encompassed a wide variety of therapies that are more or less recognized or evidence-based, and mostly unfamiliar to them.

“*The problem is that well*,* I don’t know all the complementary medicine methods. Most of the time I have no idea*.” (GP 2).

All expressed uncertainty regarding how to define CM and whether a given modality should be considered conventional, complementary, or outside these two categories (notably approaches considered “esoteric” or pseudoscientific). Some CM, like osteopathy, meditation or hypnosis, were considered almost conventional by some GPs, in the sense that they would easily or commonly recommend them to their patients or consider doing so. Some herbal medicine treatments, that are reimbursed by the mandatory health insurance, were also often considered conventional medicine.

“*Then there are perhaps some [modalities] that are [considered CM] without my knowing it*,* because it’s true that everything that has to do with relaxation*,* meditation*,* sophrology*,* yoga*,* I don’t know if it’s a [complementary] medicine (…) these are things that appeal to me a little more (…) because common sense tells me that I want to believe in it*,* and I find that it’s the continuation of my medicine. They’re not that complementary*.” (GP 10).

When asked how they would define CM, participants mostly mentioned elements that defined what it is not: not reimbursed by the mandatory health insurance, not taught in medical schools, not practiced by the GPs themselves. As one participant put it:

“*I don’t have a very precise or exhaustive definition. I’d say perhaps a little naively (…) something that’s not part of conventional*,* usual medicine. But what is really complementary? Is it a decision from health insurances to include [CM] or not [in their reimbursement plans]? Or is it more of a clinical decision? Do we expect a certain degree of validation or not? And then*,* there’s what I’m used to prescribe or practice*,* and then there’s what I’m also not familiar with*,* and which*,* by definition*,* I don’t practice*,* I don’t prescribe (…). Anyway*,* I don’t think I have a clear picture of these possible definitions*,* which may exist somewhere.*” (GP 8).

#### Openness towards CM

All but one participant spontaneously expressed being open to CM in general to a certain degree. Their attitude ranged from explicit enthusiasm to cautious support of patients’ preferences. Most GPS observed that many patients commonly use CM and that these should therefore be taken into consideration, despite their own opinion towards a particular CM.

“*It’s a reality brought to us by the patients. We realize that it’s widely used*,* that many patients find it beneficial*,* if you like. So*,* we can’t ignore this reality.*” (GP 6).

In general, participants considered CM as a useful complement to conventional medicine as it may provide alternative tools and approaches to care, thus broadening the range of treatments available to patients.

“*Sometimes you have to experiment with a lot of things until the patient finds what suits him/her. And that’s why I think it’s good that there are quite a few different ways of approaching certain problems and several different types of medicine*,* because there are some that suit some people and others that suit others.*” (GP 4).

Only one GP expressed clear reservations regarding CM. These reservations were mainly directed towards a perceived tendency to treat all CM on equal terms, although some are sustained by scientific evidence, while others are not.

“*(…) I have a fair amount of reservations (…) because I have the impression that there’s a movement going on now where if you’re not open to complementary medicine (…) you’re seen as a retrograde. Whereas I*,* who am certainly far too Cartesian*,* believe in what works*,* in what has been scientifically proven*,* if possible*,* even if by far not everything in my allopathic medicine has been scientifically proven. (…) So there you have it*,* I’ve got a problem with that*,* that these medicines are considered to be different*,* so they’re treated differently. Maybe the traditional scientific approach doesn’t apply to them*,* but I find that there is now a kind of tolerance which (…) I don’t like.*” (GP 10).

#### Skepticism of CM

Almost all GPs also expressed skepticism towards CM to a greater or lesser extent. This skepticism was either related to specific therapies or to CM therapists’ behaviors considered as problematic by GPs. When related to specific therapies, skepticism was primarily directed towards lesser-known CM, those that lacked scientific evidence or were not perceived as plausible.

“*I’m a bit blocked when it comes to things that are a bit too esoteric or things like that. When things get a bit too far out of hand. I need to have my feet firmly on the ground.*” (GP 4).

Concerns were raised regarding the insufficient training and tendency of some therapists to overstep their competencies.

“*For me*,* that’s perhaps the biggest pitfall of complementary medicine: there are quite a few therapists who know no limits. And in my profession*,* I try to really respect my limits. And I’m quite struck by the fact that some therapists have no limits. (…). So*,* what I’m trying to say is that (…) my fear is that they go beyond the spectrum of their skills. I think they really do have skills*,* but they should stop at those skills.*” (GP 11).

#### Influence of GPs’ own beliefs and experiences

GPs acknowledged that their personal beliefs and experiences had an influence on their perception of CM, whether positive or negative. For example, having personally tested a particular therapy impacted GPs’ perceptions of that therapy.

“*I think a lot of it is personal experience. As a child and young adult*,* before I started my studies*,* I was treated with homeopathy*,* and it didn’t work at all. I was treated with acupuncture and that helped a lot. I’ve never been treated with essential oils*,* but for me they’re based on plants*,* so I think that’s fine. So I think that quite a lot of my opinions are based on my personal experiences.*” (GP 4).

Similarly, some GPs underlined that they did not need to precisely understand the mechanism of this or that CM as long as it made some sense to them or at least appealed to them to some extent, as explained by one GP who was asked why he would recommend osteopathy more often than other CM.

“*Maybe because I understand [osteopathy] a bit more*,* because it seems more mechanical*,* more physical*,* closer to what I do*,* and to the way we’ve learnt to reason. It makes more sense to me. Maybe that’s why I’m going to recommend it more. I think that’s it. It appeals to me more. And I get the impression that the training is a bit clearer*,* that it’s more validated than others I know less about. (…) Acupuncture also requires training*,* it can also lead to certification and so on. Obviously. But I’m a bit less familiar with it. Maybe I don’t know as much about it*.” (GP 1).

### Recommending CM

In practice, the extent to which GPs actually recommended CM to their patients varied greatly between participants. Herbal medicine, osteopathy or acupuncture was commonly recommended by most GPs to some extent. However, for other CM, usage varied from merely discussing CM in the consultation to regularly recommending a variety of different CM. GPs’ behaviors in this context were influenced by different elements.

#### Reasons for recommending CM

Indications to use CM differed between GPs depending on which CM they were familiar with, if any. Some CM were used as first line treatments, mainly in patients presenting with benign to mild ailments (e.g., stress, anxiety, common colds, respiratory tract infections). This was particularly the case for herbal medicine, although others were also cited, such as shiatsu and yoga.

“*So yes*,* I’m quite keen on using that [herbal medicine]. Moreover*,* when the disorder isn’t severe enough. Typically for sleep disorders*,* a classic example*,* rather than prescribing benzo[diazepine]*,* whose long-term consequences we know*,* I’ll suggest herbal medicine instead. And then see with the patients whether it works or not but at least give them a trial period*.” (GP 9).

A few participants considered that CM may be an interesting option for minor conditions in cases where patients are reluctant to use conventional treatments or when they want to leave the consultation with a prescription.

Other most cited reasons for recommending CM included chronic conditions, such as chronic pain, and functional disorders. In these cases, CM was often used as a second line treatment or as a last resort when everything else has failed.

“*I’m sometimes open to it [CM] in situations where I feel that my medicine has exhausted its possibilities*,* where people have chronic pain*,* ailments that we don’t understand. I say to myself “In this case*,* we have to get out of my own medicine because (…) there’s nothing that works. We need to change the way we look at the problem”. And that means not trying another part of my medicine but trying another medicine. (…). So here I can suggest something [a CM]. (…) But that’s it*,* I think about it in these situations*,* not directly to treat back pain or pneumonia at the first consultation*.” (GP 10).

#### Barriers and facilitators

Participants identified several barriers and facilitators to recommending CM.

##### Knowledge and training

Most GPs underlined that it was easier to recommend CM for which they had at least some basic knowledge or had personally experienced.

“*And that’s why I recommend osteopathy and acupuncture. Because I’ve tried it myself and got to know it.*” (GP 4).

Conversely, lack of training or knowledge was considered a major barrier, as GPs were reluctant to recommend CM without being certain of their efficacy or when unsure about the indications.

“*The problem is that I lack training. Because I come from a scientific background. And then you hear all sorts of things. Patients come with questions (…). And frankly*,* with the need to keep up to date with the latest scientific advances [in the biomedical field]*,* I’m not yet able to do that for complementary medicine too. So sometimes I find it hard to answer questions or I find it hard to really recommend something or say ‘well*,* you see*,* scientifically it’s proven’. And that’s what I miss. (…). So I have a few notions*,* hypnosis for example for quitting smoking*,* little things like that. But because I’m not trained enough*,* it’s not yet part of the advice I give to people*.” (GP 1).

##### Efficacy

Personal beliefs regarding the efficacy of CM, as well as availability of scientific evidence were cited as important reasons for recommending CM.

“[*With] phytotherapy*,* there’s an active substance. Some of them have been studied*,* perhaps not like the latest [conventional] drugs from [pharmaceutical companies]*,* but there you go. In any case*,* there is an active substance. In my opinion*,* this is not the case with homeopathy. So*,* this is a good example of what I believe in and use. (…) So*,* I believe in phytotherapy and I use it. I don’t believe in homeopathy*,* and I don’t use it.”.* (GP 10)

##### Financial aspect

The financial aspect was seen as a barrier to recommending CM. Indeed, GPs did sometimes hesitate to recommend CM that are not covered by the mandatory health insurance to patients who do not have supplemental insurance.

“*The weak point for osteopathy is that it is not reimbursed. And there’s a clear economic barrier for some patients. (…). So I can prescribe osteopathy*,* but if they don’t go for it*,* well*,* it’s not an effective treatment. So you also have to adapt a little to the patient’s environment - and*,* ultimately*,* to their financial resources and possibilities. So sometimes we say we won’t do osteo[pathy] because the patient can’t afford it.”* (GP 8).

##### Trust

In addition, most GPs felt that they needed to be able to trust CM therapists to send them patients. GPs either trusted therapists that they personally knew, or therapists with extensive and recognized training.

“*I’m of the opinion that if I recommend hypnosis*,* it has to be done by a psychologist who specializes in hypnosis*,* because you need to have some background. I mean*,* it’s one thing to say that you can relax a little. But to really be able to do a more substantial treatment*,* you need to have good training behind you*.” (GP 2).

Most GPs acknowledged that they often lacked a network of trusted and adequately trained CM therapists to refer patients to.

“*Sometimes there are situations where I say to myself ‘maybe complementary medicine could be an asset’*,* but I don’t necessarily know who to refer to. Because then*,* for me*,* the problem - unlike perhaps a specialist*,* a cardiologist and so on - is knowing who to refer to*,* whether he or she’s really good in this field or not. It’s not the same academic training either.*” (GP 3).

To constitute a referral network for CM, participants mostly relied on discussions with and recommendations from other colleagues, and from their own experience of being treated with CM. Positive feedback from patients about their CM therapist could also lead GPs to recommend that therapist to other patients.

“*Well*,* patients tell me about their experiences [with CM]. ‘I’ve been to so and so*,* it’s done me good’. Then I say*,* ‘What does this person do?’ I’m interested. (…) In particular [there is] a therapist in [town] who does a bit of hypnosis*,* a bit of herbal medicine and a bit of energy medicine*,* all mixed. And I’ve had two or three patients who’ve come back from her with real improvements. And when someone comes in for the tenth time with the same complaint*,* and I know it’s nothing serious*,* (…) I say ‘Why not?” Then I refer them. And in the end people decide whether they want to go or not. So yes*,* I sometimes refer people like that.”* (GP 13).

### Discussing CM during the consultation

#### Approaching the subject

When asked whether they proactively enquired about their patients’ use of CM during consultations, most GPs admitted to not systematically doing so. Among them, some preferred to enquire about the type of treatments or therapies used by their patients in general.

“*I’m not asking the question specifically. I’m asking the question*,* but have you done other things? Have you thought about doing something different? That’s more like it*,* without suggesting anything. Because sometimes it comes naturally*,* sometimes not. I don’t approach it like that*,* I just approach it a little*.” (GP1).

GPs also felt that it was usually their patients who brought up the subject. Some GPs acknowledged however that their patients might not necessarily have the courage to spontaneously talk about their use of CM for fear of being judged by their GP.

“*It’s true that people [patients]*,* and this is also the problem*,* (…) don’t always dare tell us [that they use CM]. For fear*,* perhaps*,* of our judgement. They may think we’re going to judge them or tell them it’s no good. And sometimes you can tell by the way they tell us*,* that they tell us a little bit as if they were at fault*,* when in fact that’s not the case at all*.” (GP 3).

#### GP’s role in CM conversations

GPs reported that their patients seldom come with specific demands for CM but that they mostly recount what CM they use alongside their conventional treatment and sometimes ask for advice regarding these treatments. When patients did talk about their CM use, GPs expressed that they generally supported the initiatives taken by their patients or at least would not discourage them, in cases where they were skeptical about the type of CM used. Some highlighted the importance of supporting patients’ autonomy and choices and to be able to admit to not knowing about a particular CM.

“*I’m far away from this practice of medicine where we’re all-powerful*, etc.,* because I fundamentally believe that people*,* firstly*,* are free to make the choices they want. And secondly*,* they’re not my patients*,* they belong to themselves. So*,* in fact*,* I’m very happy when they talk to me about it spontaneously because it’s just part of things. And I’m quite happy to encourage them to go down that road*,* even though I always tell them that I don’t know anything about it*,* so I can’t give them any sound advice. On the other hand*,* as long as it’s the right thing for them*,* I’ll go for it*,* there’s no problem.*”. (GP 9)

Nevertheless, GPs felt it was their responsibility to accompany their patients in their treatment choices while ensuring that they do not put themselves in danger medically.

“*I find it really interesting when patients talk to us about it [CM]*,* because we have our general practitioner’s perspective*,* plus we know people*,* they’re our patients*,* and we can say ‘OK*,* he’s not taking any risks*,* he can go’ (…). On the contrary*,* we tell people ‘But be careful*,* you have high cholesterol*,* you smoke*,* you could have a heart attack. You can’t lower your cholesterol by going to the kinesiologist’*,* simple things like that. So our role (…) is more to check a little whether the patient is taking risks or not by turning to complementary medicine*,* because often it’s the patient’s choice*.” (GP 13).

GPs also sometimes tried to draw their patients’ attention to certain factors that might indicate that they are being taken advantage of, such as the costs and numbers of therapy sessions proposed by their CM therapist.

“*I also try to question them a little and stimulate their critical thinking*,* because they’re often quite vulnerable people*,* I find*,* who go a bit left and right. Some are very solid and have one or two points of reference [for CM]*,* but sometimes it’s also people with syndromes that are a bit mysterious*,* where conventional medicine doesn’t provide a precise enough answer for them. So they look for answers elsewhere*,* and sometimes the risk is that they get lost (…)*,* that they waste money*,* energy and time. I don’t think I’m being very paternalistic in this surveillance (…)*”. (GP 6)

#### Requests for laboratory analysis

Most GPs spontaneously evoked a specific situation where patients visit a CM therapist and then come to their GP with demands for a long list of laboratory analysis requested by their therapist. These demands were mostly considered as problematic.

“*Sometimes (…) we’re asked to sign laboratory orders for endless lists of analyses that I’ve never done*,* even when I’ve worked in hospital*,* even in intensive care*,* we’ve never checked these things*,* and then they should be checked… Maybe I’m reaching the limits of my knowledge*,* but sometimes I think it’s all nonsense*.” (GP 10,)

GPs would sometimes agree to prescribe those analyses, to maintain the therapeutic relationship with their patients or when they felt able to interpret the results themselves too. However, in most cases, these requests were dismissed as signing prescriptions with no clear purpose would engage GPs professional responsibility.

“*And then*,* generally speaking*,* I accept some of the analyses*,* but I accept the ones I’m capable of interpreting. Because in fact*,* if I ask for an analysis that I’m not capable of interpreting*,* and that analysis is abnormal*,* I’m in an uncomfortable position*.” (GP 11).

That same participant, along with others, also underlined GPs responsibility in controlling healthcare costs, as these analyses may be very expensive and would be reimbursed by the basic health insurance if prescribed by a physician.

“*It’s also a question of health insurances. It’s the problem of our expensive healthcare system*,* where people know they can be reimbursed. But it’s us doctors who are the guarantors of that responsibility [of signing something*,* for the patient to be reimbursed]. And we are responsible for controlling healthcare costs*,* especially GPs.*” (GP 11).

### Needs for the future

The needs expressed by the participants echo the barriers and facilitators to the recommendation of CM mentioned above, notably regarding training. As stated above, most GPs felt they lacked training or information regarding CM. They also acknowledged that it would take time to try to learn more about CM, whether through training or information seeking.

“*I’ve got patients who use complementary medicine quite a lot and in many situations it’s a help*,* it’s a plus*,* I think*,* that has its place. Now*,* personally*,* I don’t really know much about it*,* and I don’t really have the time or energy to go further and understand things. I don’t practice anything*,* I don’t have any little practice of this or that*,* nothing at all*,* apart from a little bit of herbal medicine*,* and then that’s really it*.” (GP 7).

#### Training

All GPs confirmed that teachings about CM were almost absent during their pre-graduate training. However, training needs regarding CM differed between participants. Some relied on learning by experience, through discussions with colleagues, patients or CM therapists. Some contemplated getting proper training in one CM or the other (e.g., hypnosis or phytotherapy). Finally, others advocated introductory courses, in post-graduate or continuous education, that would allow GPs to have an overview of the different CM used by the population.

“*We’re not all going to specialize in homeopathy or whatever*,* but perhaps we should have a little more basic knowledge of these different branches*,* just to know that they exist. And then*,* what can we do or achieve in terms of therapeutic results? Or what are the indications? To broaden the picture a bit*,* that could be very complementary to a GP’s practice*.” (GP 8).

A few GPs mentioned how such training would be particularly interesting for the practice of general medicine.

“*Typically*,* it [training in CM] seems to me to be of little interest to a cardiologist or a neurologist. For me*,* in general practice*,* we’re more concerned with supporting our patients and looking after their health in general. (…) And in health*,* I think that using complementary medicine can be useful. And also*,* because our role is really that of gatekeepers and guides*,* advocates and companions to our patients.*” (GP 11).

#### Information

Needs also differed regarding information about CM. Even if they sometimes sought more information when confronted with CM unknown to them, mostly through Internet searches, GPs underlined they lacked the time to systematically look for information in these cases. Information on CM was mostly gained through colleagues and patients themselves. Most GPs identified the need for a tool that would centralize information on CM and be easily accessible. GPs wished it would contain scientific information on treatment efficacy, indications and scope of action of the different CM.

“*That’s what I need. I need to know where to find information (…) that I can refer to. As for urinary tract infections*,* there are antibiotics*,* but for urinary tract infections there are also other things that work. There might be this plant or that plant and all that. And you can use it safely*,* knowing that it will still be effective. (…) That’s what I need. People who have terrible*,* raging osteoarthritis*,* and we know that a certain plant can also help*.” (GP 1).

## Discussion

Discussions around CM showed that its definition is unclear to GPs, and that a few CM were considered conventional by some. Overall, most participants expressed openness towards CM, regardless of the extent to which they actually recommended CM or the scope of their knowledge in CM. Both perceptions of and recommendations for CM were largely influenced by GPs’ personal knowledge, experiences and beliefs. CM was most often brought in the conversation by the patients themselves. GPs were generally supportive of their patients’ CM use as long as they felt it did not harm them. Finally, most GPs in our study considered that they lacked training in and information about CM.

The overall positive attitude of GPs towards CM coupled with cautiousness and skepticism when faced with the diversity of therapies and therapists was underlined in other studies [14, 16, 18, 19, 29]. What has been qualified as an ambivalent tolerance [19] may reflect the difficulty voiced by our participants of expressing a general opinion on CM, insofar as the term encompasses a multitude of different and more or less proven or familiar approaches and methods. This difficulty was also illustrated by the fact that some CM were considered by some GPs to be part of conventional medicine, while others were rejected as being too esoteric or pseudo-scientific. Other qualitative studies on the subject have also pointed out that difficulty [18, 19]. It is likely that CM that have explanatory schemes similar to those used in conventional medicine are more easily accepted. Notwithstanding, as in our study, GPs often consider CM as a complement to conventional care as it provides an additional toolbox for patient care [13, 14, 16, 17, 20]. While scientific evidence, or absence thereof, had an important impact on CM perception for some GPs, personal experiences or beliefs seemed to be equally influential factors to the formation of most GPs opinion towards a specific CM [17, 22, 29].

GPs personal experience and beliefs were also important drivers for actually recommending CM to a patient. As similarly observed by Ostermeier et al. [17], the type and number of CM that are regularly recommended by GPs in their daily practice seem to depend more often on individual preferences and experiences, as well as patients’ experiences, than on scientific evidence alone. Among our participants, those who seemed to place the most importance on scientific evidence were also those who seemed to recommend the least different types of CM. However, either herbal medicine, osteopathy or acupuncture was commonly recommended by a majority of our participants.

The three major barriers to CM recommendation identified by GPs in our study, i.e. lack of knowledge about CM (e.g., indications, scope of action), lack of a network of trusted therapists to refer patients to, and lack of insurance coverage for CM were also cited in other studies [11, 16, 18, 22, 30–32]. As suggested by some of our participants, the first two barriers could be partly overcome by improving physicians’ training on CM, a point already mentioned by Swiss GPs in a previous study [33]. Although pre-graduate training on CM has expanded in Swiss medical faculties since, the offer could be strengthened. In addition, postgraduate training, as well as continuous education in CM, could be further developed. Although not all participants expressed the need to be able to recommend CM more, courses could be designed to present an overview of at least the most common CM to allow GPs to more adequately respond to their patients’ questions. Such courses could also be an occasion for GPs to develop a network of CM therapists. This could be particularly interesting for postgraduate and continuous training in family medicine, as GPs underlined they were frequently confronted with CM in their specialty and felt it was their role to support their patients in their treatment choices and prevent them from harmful treatments. Another means to fill the GPs’ knowledge gap regarding CM would be to facilitate access to centralized and reliable information on CM and their indications, for example through a dedicated website. Another way to partly overcome those two barriers would be a stronger regulation of CM therapists practice and training. Indeed, GPs sometimes doubted the robustness of some CM therapists’ training. Such regulations may help legitimize CM and allow GPs to more confidently refer their patients, even in the absence of a formal network or strong knowledge about a particular CM [34]. Finally, lack of insurance coverage was often cited when discussing osteopathy. The status of osteopathy in Switzerland is a good example of the indetermination of what is considered CM in a given healthcare system. Indeed, osteopathy was commonly recommended by our respondents and sometimes considered as part of conventional medicine. In addition, osteopaths are recognized as healthcare professionals by the Swiss law and their training is recognized by a master’s degree. Osteopathy is also the CM that Swiss residents use the most [24]. However, osteopathy is not part of the mandatory insurance health coverage, thus impeding its full integration into the conventional healthcare system and creating inequality in access to care.

Apart from when GPs actively recommended a particular CM approach, the subject of CM was generally brought into consultation by the patients themselves. Studies have shown that physicians in general rarely enquire about their patients’ CM use and that the latter may not spontaneously disclose their CM use [35–37], notably by fear of their physician’s reaction, a risk that was also identified by our participants. Interestingly, our participants were aware of that risk but admitted to generally not proactively asking about their patient’s CM use. According to participants, patients did not usually have demands regarding CM but mostly recount their experiences with CM. Irrespective of their own opinion about the type of CM used by their patients, GPs in our study generally encouraged their patients in their care pathway. Patient-centered care, including shared decision making, patients’ autonomy and preservation of the therapeutic relationship, was privileged as long as GPs felt that their patients’ CM use would not harm them and would not engage their own professional responsibility.

The main strength of this study is to unveil how GPs in Switzerland experience and manage CM in their daily practice, thus adding to the body of knowledge on the factors influencing the use and recommendation of CM in general practice. By highlighting barriers to CM recommendation and GPs needs regarding training or information about CM, this study suggests ways in which GPs could be supported to improve communication about and facilitate referrals to CM. This study also has several limitations. First, it is possible that GPs with more positive views on CM could have more easily participated in this study than other GPs. However, the presence in our sample of a GP with clear reservations toward CM, the varying degrees of ambivalence present in the other participants, and the variety in GPs recommendation habits point to a rather diverse sample. Second, we only recruited GPs practicing in the French-speaking part of Switzerland. Our results might therefore not be transferable to the whole country. However, as our results were very similar to those conducted in other countries, notably Germany, we believe that would have found comparable results in other parts of the country. It is also noteworthy that two out of the three included cantons are bilingual (French and German), thus having cultural similarities with the rest of the country.

## Conclusions

The openness shown by GPs towards CMs in our study was nevertheless usually tinged with a certain ambivalence. GPs showed a variety of attitudes and habits regarding CM recommendation and tended to have a pragmatic approach to CM. Above all, GPs were concerned that patients should be safe and well cared for. CM use by patients is a reality, notwithstanding GPs’ own attitudes towards CM. In this perspective and given the substantial use of CM by the Swiss population, future initiatives to further integrate CM into the healthcare system should focus on providing courses and informational tools to GPs to allow them to better support and advise their patients in their treatment choices. Healthcare policies regarding CM should also take an interest in these CM which are considered almost conventional by GPs and assess whether these should be better integrated, notably through insurance coverage, to ensure equity of access for all.

## Supplementary Information

Additional file 1. Interview guide.

## Data Availability

Raw qualitative datasets analyzed during the current study are not publicly available since consent for sharing data was not granted by participants; deidentified data may be available in French from the corresponding author on reasonable request.

## Abbreviations

CM: Complementary medicine
GP: general practitioner

## References

[1] World Health organization (WHO). Traditional, complementary and integrative medicine. 2025. Available from: https://www.who.int/health-topics/traditional-complementary-and-integrative-medicine. Cited 31 Mar 2025.

[2] Lee EL, Richards N, Harrison J, Barnes J. Prevalence of use of traditional, complementary and alternative medicine by the general population: A systematic review of National studies published from 2010 to 2019. Drug Saf. 2022;45(7):713–35. 35788539 10.1007/s40264-022-01189-w PMC9296440

[3] Kemppainen LM, Kemppainen TT, Reippainen JA, Salmenniemi ST, Vuolanto PH. Use of complementary and alternative medicine in europe: Health-related and sociodemographic determinants. Scand J Public Health. 2018;46(4):448–55. 28975853 10.1177/1403494817733869 PMC5989251

[4] Steel A, McIntyre E, Harnett J, Foley H, Adams J, Sibbritt D, et al. Complementary medicine use in the Australian population: results of a nationally-representative cross-sectional survey. Sci Rep. 2018;8(1):17325. 30470778 10.1038/s41598-018-35508-y PMC6251890

[5] Ee C, Templeman K, Grant S, Avard N, de Manincor M, Hunter J. Informing the model of care for an academic integrative healthcare centre: a qualitative study exploring healthcare consumer perspectives. BMC Complement Med Ther. 2020;20(1):58. 32070328 10.1186/s12906-019-2801-4 PMC7076816

[6] Jong MC, van de Vijver L, Busch M, Fritsma J, Seldenrijk R. Integration of complementary and alternative medicine in primary care: what do patients want? Patient Educ Couns. 2012;89(3):417–22. 23031611 10.1016/j.pec.2012.08.013

[7] Ring M, Mahadevan R. Introduction to integrative medicine in the primary care setting. Prim Care Clin Off Pract. 2017;44(1):203–15. 10.1016/j.pop.2017.02.006 28501225

[8] Roberts K, Nie JB, Dowell T. From knowing silence to curious engagement: the role of general practitioners to discuss and refer to complementary and alternative medicine. J Interprofessional Educ Pract. 2017;9:104–7.

[9] Scott R, Nahin RL, Sussman BJ, Feinberg T. Physician office visits that included complementary health approaches in U.S. Adults: 2005–2015. J Integr Complement Med. 2022;28(8):641–50. 35559729 10.1089/jicm.2021.0331 PMC9467635

[10] Phutrakool P, Pongpirul K. Acceptance and use of complementary and alternative medicine among medical specialists: a 15-year systematic review and data synthesis. Syst Rev. 2022;11(1):10. 35027078 10.1186/s13643-021-01882-4 PMC8759198

[11] Schwartz MR, Cole AM, Keppel GA, Gilles R, Holmes J, Price C. Complementary and integrative health knowledge and practice in primary care settings: A survey of primary care providers in the Northwestern united States. Glob Adv Health Med. 2021;10:21649561211023377. 34249478 10.1177/21649561211023377 PMC8236774

[12] Stussman BJ, Nahin RR, Barnes PM, Ward BW. U.S. Physician recommendations to their patients about the use of complementary health approaches. J Altern Complement Med N Y N. 2020;26(1):25–33. 10.1089/acm.2019.0303 PMC6998052 31763927

[13] Ee C, Templeman K, Forth A, Kotsirilos V, Singleton G, Deed G, et al. Integrative medicine in general practice in australia: A Mixed-Methods study exploring education pathways and training needs. Glob Adv Health Med. 2021;10:21649561211037594. 34414016 10.1177/21649561211037594 PMC8369962

[14] Huber CM, Barth N, Linde K. How young German general practitioners view and use complementary and alternative medicine: A qualitative study. Complement Med Res. 2020;29(6):383–91. 10.1159/000507073 32599593

[15] Linde K, Bayer R, Gehrmann J, Jansky B. How does the role of complementary and alternative medicine in general practice differ between countries? Interviews with Doctors who have worked both in Germany and elsewhere in Europe. BMC Complement Med Ther. 2024;3(1):328. 10.1186/s12906-024-04624-w PMC11373194 39227930

[16] Liu L, Tang Y, Baxter GD, Yin H, Tumilty S. Complementary and alternative medicine - practice, attitudes, and knowledge among healthcare professionals in new zealand: an integrative review. BMC Complement Med Ther. 2021;21(1):63. 33583417 10.1186/s12906-021-03235-z PMC7882070

[17] Ostermaier A, Barth N, Linde K. How German general practitioners justify their provision of complementary and alternative medicine - a qualitative study. BMC Complement Med Ther. 2020;20(1):111. 32293399 10.1186/s12906-020-02907-6 PMC7158128

[18] Sharp D, Lorenc A, Feder G, Little P, Hollinghurst S, Mercer S, et al. Trying to put a square Peg into a round hole: a qualitative study of healthcare professionals’ views of integrating complementary medicine into primary care for musculoskeletal and mental health comorbidity. BMC Complement Altern Med. 2018;18(1):290. 30373580 10.1186/s12906-018-2349-8 PMC6206651

[19] Wardle JL, Sibbritt DW, Adams J. Primary care practitioner perceptions and attitudes of complementary medicine: a content analysis of free-text responses from a survey of non-metropolitan Australian general practitioners. Prim Health Care Res Dev. 2018;19(3):246–55. 29417914 10.1017/S1463423617000664 PMC6452961

[20] Jarvis A, Perry R, Smith D, Terry R, Peters S. General practitioners’ beliefs about the clinical utility of complementary and alternative medicine. Prim Health Care Res Dev. 2015;16(3):246–53. 24892506 10.1017/S146342361400022X

[21] Linde K, Alscher A, Friedrichs C, Wagenpfeil S, Karsch-Völk M, Schneider A. Belief in and use of complementary therapies among family physicians, internists and orthopaedists in Germany - cross-sectional survey. Fam Pract. 2015;32(1):62–8. 25381009 10.1093/fampra/cmu071

[22] Roberts K, Betts D, Nie JB, Dowell A. Navigating the path: a qualitative exploration of new Zealand general practitioners’ views on integration of care with acupuncturists. Acupunct Med J Br Med Acupunct Soc. 2021;39(4):334–42. 10.1177/0964528420929341 32631154

[23] Hahn EG. Integrative medicine and health in undergraduate and postgraduate medical education. GMS J Med Educ. 2021;38(2):Doc46. 33763531 10.3205/zma001442 PMC7958908

[24] Meier-Girard D, Lüthi E, Rodondi PY, Wolf U. Prevalence, specific and non-specific determinants of complementary medicine use in switzerland: data from the 2017 Swiss health survey. PLoS ONE. 2022;17(9):e0274334. 36103571 10.1371/journal.pone.0274334 PMC9473626

[25] Sandelowski M. Whatever happened to qualitative description? Res Nurs Health. 2000;23(4):334–40. 10940958 10.1002/1098-240x(200008)23:4<334::aid-nur9>3.0.co;2-g

[26] FMH. FMH-Ärztestatistik / Statistique médicale de la FMH. 2025. Available from: https://aerztestatistik.fmh.ch/. Cited 14 Aug 2025.

[27] Braun V. Using thematic analysis in psychology. Qual Res Psychol. 2006;3(2):77–101.

[28] Tong A, Sainsbury P, Craig J. Consolidated criteria for reporting qualitative research (COREQ): a 32-item checklist for interviews and focus groups. Int J Qual Health Care. 2007;19(6):349–57. 17872937 10.1093/intqhc/mzm042

[29] Hamilton K, Marietti V. A qualitative investigation of Australian psychologists’ perceptions about complementary and alternative medicine for use in clinical practice. Complement Ther Clin Pract. 2017;29:105–10. 29122247 10.1016/j.ctcp.2017.09.003

[30] McGuire C, Gabison J, Kligler B. Facilitators and barriers to the integration of Mind-Body medicine into primary care. J Altern Complement Med. 2016;22(6):437–42. 27148622 10.1089/acm.2016.0043

[31] Penney LS, Ritenbaugh C, Elder C, Schneider J, Deyo RA, DeBar LL. Primary care physicians, acupuncture and chiropractic clinicians, and chronic pain patients: a qualitative analysis of communication and care coordination patterns. BMC Complement Altern Med. 2016;16(1):30. 26810302 10.1186/s12906-016-1005-4 PMC4727288

[32] Roberts K, Dowell A, Nie JB. From research to practice: building bridges to enhance interprofessional communication between general practitioners and acupuncturists. J Commun Healthc. 2020;13(3):234–44.

[33] Schneider S, Graz B, Rodondi PY, Bonvin E. Attitudes des médecins généralistes envers les médecines complémentaires et besoins de formation. Résultats d’une enquête Suisse. Pédagogie Médicale. 2014;15(2):157–60.

[34] Carè J, Steel A, Wardle J. Stakeholder attitudes to the regulation of traditional and complementary medicine professions: a systematic review. Hum Resour Health. 2021;19(1):42. 33781297 10.1186/s12960-021-00579-y PMC8008552

[35] Foley H, Steel A, Cramer H, Wardle J, Adams J. Disclosure of complementary medicine use to medical providers: a systematic review and meta-analysis. Sci Rep. 2019;9(1):1573. 30733573 10.1038/s41598-018-38279-8 PMC6367405

[36] Jou J, Johnson PJ. Nondisclosure of complementary and alternative medicine use to primary care physicians: findings from the 2012 National health interview survey. JAMA Intern Med. 2016;176(4):545–6. 26999670 10.1001/jamainternmed.2015.8593

[37] Shelley BM, Sussman AL, Williams RL, Segal AR, Crabtree BF. They don’t ask me so I don’t tell them’: Patient-Clinician communication about traditional, complementary, and alternative medicine. Ann Fam Med. 2009;7(2):139–47. 19273869 10.1370/afm.947 PMC2653970

[38] Fedlex. SR 810.30 - Federal Act on research involving human beings. 2011. Available from: https://www.fedlex.admin.ch/eli/cc/2013/617/en. Cited 10 Mar 2025.

[39] World Medical Association. World medical association declaration of helsinki: ethical principles for medical research involving human subjects. JAMA. 2013;310(20):2191–4. 24141714 10.1001/jama.2013.281053

