# Supplementary material for: “It's a reality brought to us by the patients”: a qualitative study on general practitioners’ views on and experience with complementary medicine in Switzerland
Source: BMC Prim Care. 2025 Oct 17;26:320. doi: 10.1186/s12875-025-02999-4 (PMC12532388; doi:10.1186/s12875-025-02999-4)
Supplement: Supplementary file 1 — Additional file 1. Interview guide. [file 12875_2025_2999_MOESM1_ESM.pdf]

Themes	Questions	Follow-up questions
**CM in general practice**	What do you think about the use of complementary medicine practitioners in family medicine?<br><br>Can you describe/tell me about your use or non-use of complementary medicine (CM)?<br>- *(If used)* In what situations do you most often use/prescribe/recommend it?<br><br>- (*If not used*) Why ?<br><br>Is there a demand from your patients for CM?<br>If yes, why do they ask for it (what types of pathologies/health problems)?<br><br>If no, why do you think you don't receive such requests?<br><br>Do you bring up CM during your consultations?<br>If yes, how?<br><br>Can you describe one consultation related to CM from the past year?<br>(*A situation that was representative for you and one that was completely unexpected*)	-What difficulties do you encounter when recommending/prescribing CM?<br>-What elements facilitate recommending/prescribing CM?<br>-To what extent do you feel comfortable using/prescribing/recommending CM?<br><br>-Chronic diseases, therapeutic uncertainty, desire to prescribe something to patients<br><br>-Lack of knowledge, lack of network<br><br>-To what extent do you feel comfortable talking about CM with patients who ask about it?<br>-What do you lack in order to respond to these requests in a way you find satisfactory?<br>-What helps you to respond to these requests in a way you find satisfactory?

**Training**	What do you think of the training you received during your medical studies about CM?	- Quantity and quality of training<br>- Sources of information on CM<br>- Interest in further training
**Collaboration with CM therapists**	How would you describe collaboration with CM therapists?<br>Can you give me an example?<br><br>To what extent would you like to develop collaborations with CM therapists?	-What are the obstacles or facilitators in your collaboration with CM therapists?<br>-Do you ever receive reports from CM therapists consulted by your patients?<br>- To what extent do you know to whom you could refer a patient wishing to consult a CM therapist?
